# Expanding the Utilization of Formalin-Fixed, Paraffin-Embedded Archives: Feasibility of miR-Seq for Disease Exploration and Biomarker Development from Biopsies with Clear Cell Renal Cell Carcinoma

**DOI:** 10.3390/ijms19030803

**Published:** 2018-03-09

**Authors:** Philipp Strauss, Hans-Peter Marti, Christian Beisland, Andreas Scherer, Vegard Lysne, Sabine Leh, Arnar Flatberg, Even Koch, Vidar Beisvag, Lea Landolt, Trude Skogstrand, Øystein Eikrem

**Affiliations:** 1 Department of Clinical Medicine, University of Bergen, 5021 Bergen, Norway; philipp.strauss@uib.no (P.S.); hans-peter.marti@uib.no (H.-P.M.); christian.beisland@uib.no (C.B.); sabine.leh@helse-bergen.no (S.L.); even.koch@uib.no (E.K.); lea.landolt@uib.no (L.L.); 2 Department of Medicine, Haukeland University Hospital, 5021 Bergen, Norway; 3 Department of Urology, Haukeland University Hospital, 5021 Bergen, Norway; 4 Spheromics, 81100 Kontiolahti, Finland; andreas.scherer@spheromics.com; 5 Institute for Molecular Medicine Finland (FIMM), University of Helsinki, 00100 Helsinki, Finland; 6 Department of Clinical Science, University of Bergen, 5021 Bergen, Norway; vegard.lysne@uib.no; 7 Department of Pathology, Haukeland University Hospital, 5021 Bergen, Norway; 8 Department of Clinical and Molecular Medicine, Norwegian University of Science and Technology, 7491 Trondheim, Norway; arnar.flatberg@ntnu.no (A.F.); vidar.beisvag@ntnu.no (V.B.); 9 Department of Biomedicine, University of Bergen, 5021 Bergen, Norway; trude.skogstrand@uib.no

**Keywords:** microRNA/miRNA, miR-155, clear cell renal cell carcinoma/ccRCC, formalin-fixed paraffin-embedded/FFPE, next generation sequencing/NGS

## Abstract

Novel predictive tools for clear cell renal cell carcinoma (ccRCC) are urgently needed. MicroRNAs (miRNAs) have been increasingly investigated for their predictive value, and formalin-fixed paraffin-embedded biopsy archives may potentially be a valuable source of miRNA sequencing material, as they remain an underused resource. Core biopsies of both cancerous and adjacent normal tissues were obtained from patients (*n* = 12) undergoing nephrectomy. After small RNA-seq, several analyses were performed, including classifier evaluation, obesity-related inquiries, survival analysis using publicly available datasets, comparisons to the current literature and ingenuity pathway analyses. In a comparison of tumour vs. normal, 182 miRNAs were found with significant differential expression; miR-155 was of particular interest as it classified all ccRCC samples correctly and correlated well with tumour size (*R*^2^ = 0.83); miR-155 also predicted poor survival with hazard ratios of 2.58 and 1.81 in two different TCGA (The Cancer Genome Atlas) datasets in a univariate model. However, in a multivariate Cox regression analysis including age, sex, cancer stage and histological grade, miR-155 was not a statistically significant survival predictor. In conclusion, formalin-fixed paraffin-embedded biopsy tissues are a viable source of miRNA-sequencing material. Our results further support a role for miR-155 as a promising cancer classifier and potentially as a therapeutic target in ccRCC that merits further investigation.

## 1. Introduction

Kidney cancer is one of the most common cancers in the Western world, accounting for 2–3% of all adult malignancies worldwide [1,2], and its incidence rate is projected to accelerate [3]. Over 50% of kidney cancers are of the clear cell renal cell carcinoma (ccRCC) subtype [2,4,5], which is often characterized by an inactivation of the von *Hippel-Lindau* gene and arises from the epithelium of the proximal tubule [2,4,6]. The majority of cases are discovered at an advanced stage [4,7], with even small tumours exhibiting metastatic potential [8,9]. Neither chemotherapy, targeted therapy, nor radiotherapy currently represent effective avenues of treatment for the advanced stages, with radical surgery presently being the best option [2,10,11,12]. For these reasons, there is an unmet need to discover biomarkers of ccRCC [13]. It is therefore of great importance to enhance our understanding of the pathophysiology of ccRCC, as this will enable us to develop novel diagnostic, therapeutic and predictive measures.

Micro-RNAs (miRNAs) have increasingly emerged not only as biomarkers and predictive tools but also as therapeutic targets [14,15,16,17,18,19,20]. miRNAs are single stranded, non-coding RNA molecules with lengths of 19–22 nucleotides [21,22]. They are heavily involved in post-transcriptional regulation of mRNA, making them ideal candidates both as biomarkers and as tools for diagnosis and therapy [20,23,24,25]. 

Several techniques are currently employed to study miRNAs. One of these techniques is next generation sequencing (NGS), which offers unique options for detecting novel miRNA transcripts. NGS can also quantify expression levels of miRNAs precisely [26,27]. While using fresh-frozen (FF) samples is more common in miRNA research, collecting a sufficient number of samples can be time-consuming, especially when a long follow-up is desirable. In contrast, formalin fixation and paraffin embedding (FFPE) has been used as an easily accessible method for several decades. Consequently, there are large archives of FFPE biopsies, with a wealth of information on the associated patients’ subsequent clinical development. These archives remain an underused resource, as NGS of FFPE biopsies was previously thought to yield results of insufficient quality. However, results comparable to those of FF samples have been obtained, even using highly degraded FFPE samples [28,29,30,31] Several investigations of the miRNA profile of ccRCC have been performed [7,32], as have studies on FFPE specimens [14,33]. However, to the best of our knowledge only the work of Weng et al. has investigated the miRNA profile of ccRCC with NGS of samples derived from FFPE [28] Although novel, the work of Weng et al. included only three cases of ccRCC and their findings, therefore, require further investigation and validation. The primary aim of this study was to validate the analysis of stored FFPE ccRCC biopsies with NGS in a larger cohort than Weng et al. [28]; and, secondly, to examine the difference between ccRCC and normal tissues with regard to miRNA levels. 

## 2. Results

### 2.1. RNA Yield and RNA Quality

Sufficient RNA for NGS was extracted from all enrolled participants (Table 1), with an average RNA yield of 1069 ng per sample. The mean DV200 value was 54% (with a 95% confidence interval (CI) of 48–61%), which was of sufficient quality for NGS [30].

### 2.2. miRNA Expression Analysis and Data Visualization

**Figure 1:**
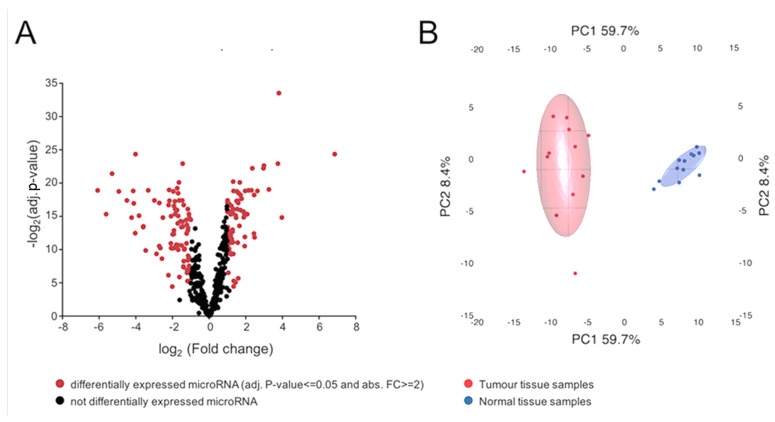
(**A**) Volcano plot of all detected miRNAs. From a total of 730 detected miRNAs, 182 were differentially expressed. Of all detected miRNAs, 423 were upregulated and 307 were downregulated in tumor samples. Among the differentially expressed miRNAs, 103 were downregulated and 79 were upregulated; (**B**) principal component analysis of the 182 differentially expressed miRNAs (those with an adjusted *p*-value < 0.05 and absolute fold change > 2). Samples segregate according to their diagnosis, with larger variation found amongst tumour tissue samples, as shown by the larger spread in that group, particularly along principal component 2.

Based on the expression filter, a total of 730 miRNAs were detected with statistical confidence, amongst which 423 were overrepresented and 307 were underrepresented in tumour samples, and 182 showed significant differential expression between the tumour and normal samples. Amongst the differentially expressed genes, 103 were downregulated in the tumour samples and 79 were upregulated. The volcano plot in Figure 1A displays the entire study population divided by detected miRNAs that were differentially expressed (red) or not (black).

The most strongly upregulated miRNA was miR-122-5p (FC = 116.04). The most strongly downregulated miRNA was miR-184 (FC = −67.61). The 20 most differentially expressed miRNAs are displayed in Table 2, sorted by the abs. FC between tumour and normal tissues. 

### 2.3. Evaluation of Selected miRNAs as Potential Classifiers

**Figure 2:**
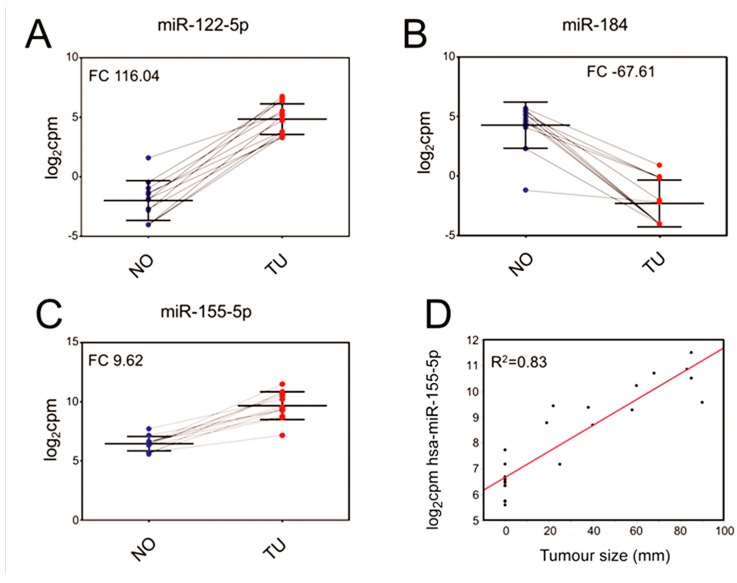
(**A**) Scatterplots of the expression of miR-122-5p (adj. *p*-value 4.68× 10^−8^); (**B**) miR-184 (adj. *p*-value 2.05 × 10^−6^) and (**C**) miR-155-5p (adj. *p*-value 1.86× 10^−6^) to classify samples as either tumour (TU, in red) or normal (NO, in blue). Each dot represents the result from one sample. Normal and tumour samples originating from the same donor are connected by lines; (**D**) displays the correlation of miR-155-5p expression and tumour size, which results in a linear regression with *R*^2^ = 0.83.

miR-184, miR-155-5p and miR-122-5p were evaluated as classifiers, i.e., to separate tumours from normal tissue (Figure 2A,C). miR-122-5p and miR-184 were selected due to having the two highest overall abs. FC., while miR-155-5p was selected due to findings in other analyses in this investigation. The best separation of samples was achieved using miR-122-5p (Figure 2A), with a log_2_CPM of 2. In this way, every sample was correctly classified as either tumour or normal tissue. Similarly, a log_2_CPM cut-off of 1 for miR-184 (Figure 2B) resulted in one normal sample being classified incorrectly, while all others were correctly classified as either tumour or normal tissue. 

A log_2_CPM cut-off of 7 for miR-155-5p (Figure 2C) incorrectly classified two normal samples but correctly classified all others. 

### 2.4. Correlation of miRNA with Tumour Size

miR-155-5p (*R*^2^ = 0.83), miR-10b-5p (*R*^2^ = 0.80), miR-361-3p (*R*^2^ = 0.78) and miR-10b-3p (*R*^2^ = 0.78) showed the best correlation with tumour size. Only the results for miR-155-5p (Figure 2D) are displayed.

### 2.5. Survival Analysis

**Figure 3:**
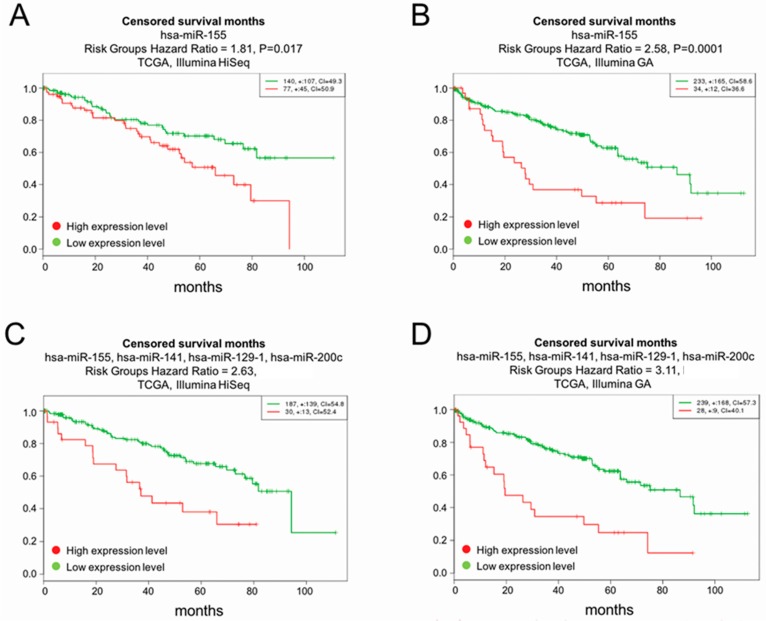
(**A**,**B**) Overall survival analysis of miR-155; (**A**) displays the findings from the Hiseq dataset, while **(B**) shows the findings from the Illumina GA dataset; (**C**) overall survival analysis of miR-155, miR-141, miR-129, miR-200c in the HiSeq dataset (*p* = 6.6 × 10^−4^). (**D**) The most statistically significant findings were made using a multivariate approach with 4 miRNAs combined in the Illumina GA dataset (*p* = 1.27 × 10^−5^).

Based on the high absolute fold changes between normal and tumour samples and correlation with tumor size, miR-155-5p, miR-122-5p, miR-184 and miR-514 were tested for survival analyses as a single marker. Using miR-155-5p as a single marker, the most significant finding in the Cancer Genome Atlas (TCGA) Illumina GA dataset was obtained with miR-155-5p (Figure 3B), with *p*-value = 0.0001 and hazard ratio (HR) = 2.58 (CI: 1.59–4.17). In the TCGA Illumina HiSeq dataset (Figure 3A), miR-155-5p showed *p*-value = 0.0175, HR = 1.81 (CI: 1.11–2.96). The second most significant miRNA was miR-122-5p (not shown in Figure 3). In the Illumina GA dataset, the analysis of miR-122-5p resulted in *p*-value = 0.02407 and HR = 1.7 (CI: 1.07–2.69). In TCGA Illumina Hiseq, the findings for miR-122-5p were not statistically significant: *p*-value = 0.07513 and HR = 0.63 (CI: 0.38–1.05). The most statistically significant findings were made using a multivariate approach with 4 miRNAs combined in the Illumina GA dataset (Figure 3D). Survival analysis of miR-155, miR-141, miR-129, miR-200c in combination gave a HR of 3.11 (CI: 1.87–5.18) and (*p* = 1.27 × 10^−5^). The same four miRNAs gave a HR of 2.63 (CI = 1.51–4.6), *p* = 6.6 × 10^−4^, in the TCGA Illumina Hiseq dataset. See Figure 3 for more details. The authors would like to point out that the survival analyses were not intended to be exhaustive and, therefore, the complete adherence to the REMARK guidelines [34] goes beyond the scope of this investigation. 

**Figure A1:**
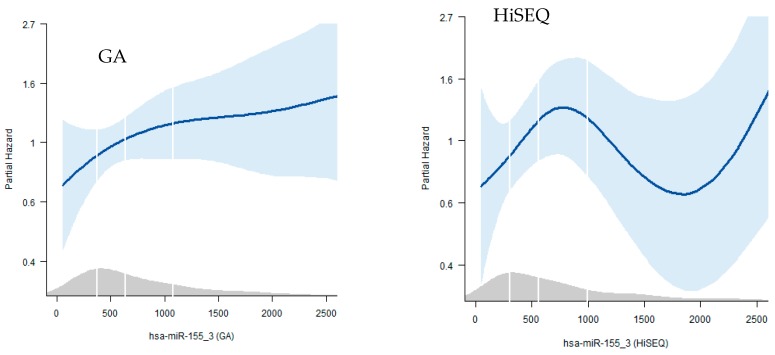
The visual representation of the Cox multivariate analyses, showing the continuous relationship between the expression of mir-155 and risk, adjusted for age, sex, histological grade and cancer stage. The white lines indicate the 1st, 2nd, 3rd and 4th quartile of gene expression. The figure on the left was generated with data from the GA dataset, the figure on the right was generated with data from the HiSeq dataset.

Nonetheless additional, multivariate cox regression analyses with estimated hazard ratios were performed, using the levels of miR-155-5p, miR-141, miR-129-1 and miR-200c. Additionally, age, gender, stage and tumour histological grade were considered. Results are displayed in Table 3. Only the results from miR-155 are shown. The accompanying figures are shown in the Appendix A as Figure A1. The results were statistically significant comparing the lowest quartile with the third and fourth quartile for model 1 with age and sex together with the expression values of miR-155 for the GA dataset only. In the complete model where also cancer stage and histological grade were considered, the results were not statistically significant. Thus, combining miR-155 expression data and standard clinical parameters in a Cox hazard model did not benefit the survival prediction. The excel-file containing the clinical and histopathological data from the TCGA investigation has been uploaded as supplementary information.

### 2.6. Correlation of miRNA Abundance to Body Mass Index (BMI)

Since obesity has been linked to ccRCC [35,36], the dataset was investigated for correlations between BMI and the expression level of various miRNAs. The best correlation was seen for miR-10a-3p (*R*^2^ = 0.68). Other high-ranking candidates were miR-10a-5p (*R*^2^ = 0.65) and miR-487a-3p (*R*^2^ = 0.64). Several comparisons between tumour and normal tissues from different BMI groups were then performed (Table A1). The miRNA with the strongest difference between tumour and normal samples in patients with high BMI was miR-122-5p (abs. FC: 280), whereas the corresponding miRNA in patients with low BMI was miR-184 (abs. FC: 310), both of which were statistically significant. 

### 2.7. Pathway Analyses

**Figure A2:**
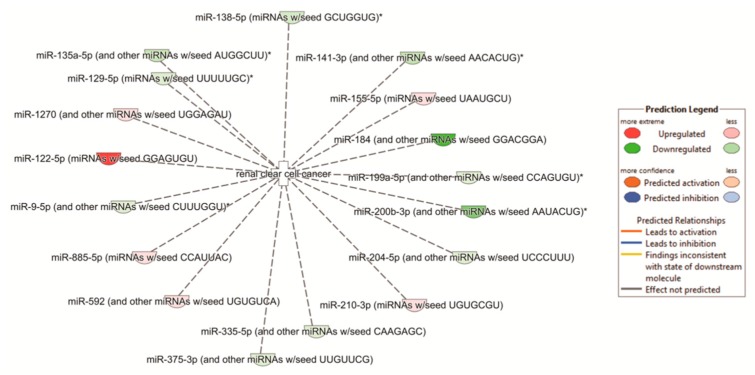
A visual representation of the “renal clear cell cancer” network and how our data relates to this. The *p*-value was 2.84 × 10^−^^4^.

To determine which biological pathways were overrepresented by the differentially expressed miRNAs, we performed pathway analysis. The integration of the present miRNA data with previously published mRNA data from the same patient cohort [30] identified the Th2 pathway as the most affected (*p*-value = 6.23 × 10^−11^). Additionally, all other top pathways were related to either immunology or fibrosis (Table 4). Using the previously published data of mRNA obtained from the same patients, another pathway analysis was performed using the differentially expressed miRNA genes and their differentially expressed mRNA targets. The top five upstream regulators, each with *p*-values of 1.12 × 10^−14^ or less, are shown in Table 4. The network analysis of “renal clear cell cancer” showed a *p*-value of 2.84 × 10^−4^. The specific up- and down-regulation of various parts of the network are displayed in Figure A2. 

### 2.8. Confirmation of Differentially Regulated miRNA

In an investigation comparable to ours, Osanto et al. [27] used FF samples from a similarly sized cohort to identify miRNAs in ccRCC with NGS technology. Of Osanto’s top 20 differentially expressed miRNAs, 14 were detected amongst the differentially expressed miRNAs from the present study, with a 70% overlap. Of those 14 miRNAs, 5 were amongst the top 20 differentially expressed miRNAs from this dataset. The direction of the FC was the same for any given miRNA found in both datasets. Both of these original investigations were compared to an inquiry with an even larger sample pool, using a dataset from TCGA and two previously published cohorts [18]. Of the 17 miRNAs described by Shu et al. [37], 16 were found amongst the differentially expressed miRNAs from this investigation (94% overlap). The direction of the FC was the same for any given miRNA. Four of the 17 were amongst the top 20 miRNAs from our dataset (Table A2).

## 3. Discussion

In this report we investigated the use of FFPE biopsies for miR-seq by examining the ccRCC miRNA profile. The use of miRNAs as tumor classifiers have been reported and confirmed in many previous studies [7,25,27,37,38]. However, we are the first to extend the previous, but more limited, findings of Weng et al. on NGS of FFPE samples [28], by including four times as many patients. Still, a total patient number of 12 and total sample number of 24 is also a relevant limitation of this investigation. The limited number of patients in this investigation is also the reason why we used the TCGA dataset for the survival analyses. In this study, we extracted both quantitatively and qualitatively sufficient RNA for miRNA sequencing from all 24 FFPE samples. However, successful sequencing does not preclude the possibility of a bias inherent to FFPE samples when compared to FF samples. In our previous investigation of this issue [30] we found a correlation between the differentially expressed mRNA found in paired FF vs. FFPE biopsies of *R*^2^ = 0.96, while Weng et al. [28] found an miRNA correlation of *R*^2^ = 0.95–0.98. In addition to the comparisons with the findings of both Osanto [27] and Shu [37], this supports the absence of any obvious bias. This study, therefore, presents further evidence that FFPE samples are a viable source for miRNA sequencing of ccRCC samples. FFPE biopsy archives remain an underused resource for developing patient stratification and treatment tools; however, we believe that our present findings can help to further unlock these archives. To further demonstrate the usefulness of the current miR-seq data, we performed additional analyses linking our investigation to more biologically relevant examinations. 

The combination of the results of the classifier analysis, the matching of miRNA abundance to tumour size, and the survival analyses, makes us regard miR-155 as the most interesting miRNA highlighted in this investigation. The Cox multivariate analyses performed on the GA dataset resulted in a close to linear increase of risk with increasing levels of miR-155. However, once correcting for age, sex, cancer stage and histological grade, miR-155 was not a statistically significant survival predictor. If the results of our survival analysis even approximately translate to the clinical setting, it would indicate that patients with high levels of miR-155-5p are almost three times as likely to die over a given period of time. One possible explanation for why an elevated expression of miR-155-5p is correlated with poorer overall survival is that increased expression of miR-155-5p also correlated with larger tumours in this dataset, which in turn has been linked to lower survival [39]. miR-155-5p was one of only two miRNAs found amongst the top 20 miRNAs in our dataset along with those from Osanto and Shu [27,37]. Previously, miR-155-5p has been investigated in a wide range of settings. In ccRCC, miR-155 was first identified as a target of interest while profiling the differences between various cancers and normal kidney tissues [38,40]. miR-155 has also been evaluated as a possible distinguisher of metastatic and non-metastatic cancers, both for untreated [41] and sunitinib-treated patients [42]. In the latter study, decreased levels of miR-155 were significantly associated with increased time of tumour progression. To explain the underlying mechanism of miR-155-5p, E2F2 has been proposed as a possible target [43]. Some of the predicted functions of miR-155-5p include the inhibition of proliferation, migration and induction of apoptosis by upregulating BACH1 in renal cancer cells [44]. Suppression of miR-155 also significantly inhibits the proliferation, colony formation, migration and invasion of ccRCC cells, while inducing G1 arrest and apoptosis and upregulating FOXO3a [45]. However, we are the first to propose miR-155-5p expression as an important predictor of tumour size and one of the first to examine its relation to survival. Although several interesting findings for miR-155 has been demonstrated, once correcting for age, sex, cancer stage and histological grade the predictive value of overall survival in the TCGA dataset statistical significance was lost.

We were unable to detect significant changes in the expression of miRNAs in the tumours of patients with different BMIs, at least in our limited dataset. Shu et al. suggested that miR-200a-3p, miR-200b-3p, miR-200c-3p, miR-210-3p, miR-204-5p and miR-30a-5p may all be obesity-related and were amongst their top 17 miRNAs [37]. In our results, all these miRNAs were differentially expressed and changed in the same direction as reported in Shu’s dataset. However, none of them were amongst the 4 most differentially regulated miRNAs in any analysis displayed in Table A1, nor were they amongst the miRNAs with the strongest correlation with BMI. 

Senbabaoglu et al. previously described the importance of the Th2 pathway in ccRCC [46]. Consistently, in our combined miRNA and mRNA dataset, the Th2 pathway was most upregulated. Thus, this pathway may represent a novel therapeutic target for ccRCC.

Many of our top miRNAs, e.g., miR-122 [47], miR-200c [48] and miR-210 [49], have previously been linked to the epithelial–mesenchymal transition (EMT). In support of this, Lorens et al. previously proposed EMT to be a central inducer of Axl expression [50]. More recently, downregulation of miR-217 has been linked to HIF-1α/AXL signalling via the suppression of HIF-1*α* protein levels [51]. In the present dataset, miR-217 was amongst the most differentially expressed miRNAs. In the previously published data from this cohort, several mesenchymal markers were screened [30], revealing an over-representation of vimentin (VIM), endothelin 1 (EDN1), fibronectin 1 (FN1), and transforming growth factor-β (TGF β1). Additionally, the epithelial markers epithelial cell-adhesion molecule (EPCAM) and E-cadherin (CDH1) were under-represented. Grainyhead-like 2 (GRHL2), a transcription factor that has been shown to inhibit EMT, was approximately 10-fold downregulated [30]. This connection to EMT is further strengthened by the top upstream regulators found in our pathway analysis, in which the top five deregulated upstream regulators were all linked to EMT: interferon gamma [52], tumour necrosis factor [52,53], lipopolysaccharide [54], TGFB1 [55], and beta-estradiol [56]. 

The findings presented here require functional validation, especially if we wish to further investigate these miRNAs as targets in the development of novel therapies. Additionally, the use of serum samples for detecting miR-155-5p requires further investigation, and it must be determined whether those results match our findings from solid biopsies. 

## 4. Materials and Methods

### 4.1. Participants

Twelve ccRCC patients undergoing either partial (*n* = 4) or radical (*n* = 8) nephrectomy at Haukeland University Hospital were selected consecutively. None of the patients had undergone previous treatment. Further details of the study population have been reported previously [57]. In short, pT tumour stages of T1a/b (*n* = 7), T2a/b (*n* = 2) and T3a/b (*n* = 3) were included. The mean age was 56.9 ± 6.8 years. Five of the 12 participants were males. Tumour sizes varied from 15 mm to 117 mm with an average of 46 mm. All clinical information was acquired from the patients’ medical records and our own in-house renal cancer registry. Leibovich, Fuhrmann and tumour-node-metastasis (TNM) scoring was performed in accordance with the established criteria [10], based on routine workup. Body mass index (BMI) groups were established as follows: BMI low: 19–23 (*n* = 4) and BMI high: 28–44 (*n* = 4). Additional patient characteristics are displayed in Table 1. The ethics committee of Western Norway approved this study on 06/06/2005 (REC West No. 78/05). All participants provided informed consent. 

### 4.2. Kidney Biopsies and RNA Extraction

All core biopsies were obtained with 16-gauge core biopsy needles. Both tumour and tumour-adjacent normal samples were taken from each patient in the operating room at the time of surgery, immediately following tumour removal. Tumour and tumour-adjacent normal tissues were identified visually at the time of sampling and subsequently stored as FFPE tissue. Histological confirmation was then performed by an experienced pathologist. RNA was extracted using a miRNeasy FFPE kit (Qiagen, Venlo, The Netherlands). All extractions were performed as previously established [57,58] and in accordance with the manufacturer’s instructions. Eight 10 µm sections were used from each FFPE block. The quality and quantity of the extracted RNA were measured with a NanoDrop spectrophotometer (Nano Drop Technologies, Wilmington, DE, USA) and an Agilent RNA 6000 Nano Kit with a 2100 Bioanalyzer instrument (Agilent Technologies, Santa Clara, CA, USA). The DV200 metric, which is the percentage of fragments >200 nucleotides in length [59], was computed from a standard smear analysis on the 2100 Bioanalyzer instrument as an indicator of quality.

DV200 values of as low as 30% have been reported in the creation of RNA libraries [60]. DV200 was used instead of the RIN number because RIN (RNA integrity number) is not a reliable parameter of RNA quality in degraded FFPE samples. RIN is also an unreliable predictor of cDNA library output for FFPE-extracted RNA compared to the DV200 metric [61]. Following RNA extraction, samples were stored at −80 °C.

### 4.3. Small RNA Library Preparation and Sequencing

Prior to library preparation, sample RNA concentrations were measured with a Qubit RNA HS Assay Kit on a Qubit 2.0 fluorometer (Thermo Fisher Scientific, Waltham, MA, USA).

The sequencing libraries were generated with a TruSeq small RNA library kit (Illumina, USA, Inc., San Diego, CA, USA) in accordance with the manufacturer’s protocol, using 1 µg total RNA as starting materials for 20 samples and slightly less for the remaining 4 samples. Prior to sequencing, the libraries were normalized, pooled and size selected (145–160 bp) before clean up. Finally, the pooled libraries were normalized, and 2.2 pM was subject to clustering on the instrument’s flow cell. The clustering and sequencing (50 cycles) were performed on a NextSeq500 instrument, in accordance with the manufacturer’s instructions (Illumina, Inc., San Diego, CA, USA). FASTQ files were created with bcl2fastq 2.18 (Illumina, Inc., San Diego, CA, USA). Data will be made available through the Gene Expression Omnibus repository. 

### 4.4. Statistics and Next Generation Sequencing (NGS) Data Processing

Fastq files were adapter filtered using fastq-mcf and miRNA expression values were generated with miRDeep2 using gene definitions from miRBase 21. An empirical expression filter was applied, to only retain genes with more than 3 counts per million (cpm) in more than 8 samples per dataset. Comparative analysis was done using the voom/limma R-package [62,63] (Available online: www.Bioconductor.org) (R Bioconductor version 3.4). Differential gene expression was defined as a Benjamini–Hochberg-adjusted *p*-value ≤ 0.05, and an absolute fold change (abs. FC) ≥ 2. Pathway analysis was performed with Ingenuity Pathway Analysis (Qiagen, Redwood City, CA, USA; version 27216297). The Ingenuity Knowledge Base was used as a reference dataset.

Canonical pathways were sorted by their smallest Benjamini-Hochberg-adjusted *p*-value.

Classifier analysis was then performed with the KNN Validation package in GenePattern (Available online: http://www.broadinstitute.org/cancer/software/genepattern). Euclidean distance was used as distance measure, where three neighbours were considered. Additional analyses and data visualization was performed with JMP Pro 11 (Available online: www.sas.com), and Graphpad Prism 6 (Available online: www.graphpad.com).

### 4.5. Survival Analysis

To analyse survival rates, The Cancer Genome Atlas (TCGA) NGS data were analysed using the SurvExpress platform (Available online: http://bioinformatica.mty.itesm.mx:8080/Biomatec/Survmicro.jsp). All features were averaged per sample. Statistical analyses were performed using the Kaplan Meier log-rank test and Cox proportional hazard regression to determine the relationship between gene expression and survival time. High- and low-risk groups were categorized according to significantly different survival rates. Two ccRCC datasets were used: the renal clear cell carcinoma (Illumina GA) TCGA dataset (*n* = 267), and the renal clear cell carcinoma (Illumina HiSeq) TCGA dataset (*n* = 217) (Figure 3). Mir-155 was also tested in a multivariate Cox-regression model with age, sex, histological grade and cancer stage (Table 3). This is also plotted graphically demonstrating the partial hazard for a given expression value as a continuous variable (Figure A1). Multivariate Cox regression analyses were performed using R software version 3.4.30 (R foundation for Statistical Computing, Vienna, Austria; R-Studio version 1.1.383; packages tidyverse and survival).

## 5. Conclusions

FFPE biopsies are an entirely viable source of material for miRNA analyses. FFPE biopsy archives remain an underused resource for molecular analyses. miR-seq from FFPE tissues demonstrated the potential of finding candidate markers once larger FFPE datasets are used. We found that miR-155 has a high correlation with tumor size as well as demonstrating its potential as a classifier in ccRCC. We believe that our present findings can help to further unlock these FFPE archives.

## Figures and Tables

**Table 1:** Patient characteristics at the time of surgery.

Patient Number	Age (Year)	Gender	BMI	Nephrectomy Type	eGFR	TNM-Stage	Size (mm)	Fuhrman Grade	Leibovich Score	Stage
39	71	Male	25	Radical	59	pT3AcN0cM0	90	4	8	III
44	74	Female	23	Radical	>60	pT3AcN0cM0	58	4	4	III
46	53	Female	24	Partial	>60	pT1AcN0cM0	38	1	0	I
50	72	Female	19	Radical	>60	pT1BcN0cM0	68	2	3	I
53	46	Female	44	Radical	>60	pT2AcN0cM0	83	2	3	II
55	44	Female	23	Radical	>60	pT3AcN0cM0	85	3	5	III
57	63	Female	28	Radical	>60	pT1AcN0cM0	25	2	0	I
59	52	Female	29	Partial	>60	pT1AcN0cM0	40	2	0	I
63a	55	Male	28	Partial	>60	pT1AcN0cM0	19	3	1	I
63b	44	Male	20	Partial	>60	pT1AcN0cM0	22	2	0	I
64	52	Male	26	Radical	>60	pT1BcN0cM0	60	3	4	I
65	57	Male	24	Radical	>60	pT2AcN0cM0	85	3	5	II

BMI: body mass index, eGFR: estimated glomerular filtration rate, measured in (mL/min/1.73 m^2^) TNM: tumor node metastasis performed according to the European Association of Urology (EAU) guidelines on renal cell carcinoma; 2014 update [10], cN0: clinically assessed negative lymph nodes, cM0: clinically assessed no metastasis.

**Table 2:** The 20 miRNAs with the highest absolute fold change. TU: tumour, NO: normal.

Mature microRNA	Precursor microRNA	Fold Change (TU/NO)	*p*-Value	Adjusted *p*-Value
hsa-miR-122-5p	hsa-miR-122	116.04	2.60 × 10^−10^	4.68 × 10^−8^
hsa-miR-184	hsa-miR-184	−67.61	8.22 × 10^−8^	2.05 × 10^−6^
hsa-miR-891a-5p	hsa-miR-891a	−49.12	3.95 × 10^−6^	2.43 × 10^−5^
hsa-miR-200c-3p	hsa-miR-200c	−39.12	6.84 × 10^−9^	3.59 × 10^−7^
hsa-miR-141-5p	hsa-miR-141	−30.31	1.25 × 10^−7^	2.29 × 10^−6^
hsa-miR-514a-3p	hsa-miR-514a-2	−22.31	4.82 × 10^−7^	5.87 × 10^−6^
hsa-miR-216b-5p	hsa-miR-216b	−18.72	6.35 × 10^−6^	3.50 × 10^−5^
hsa-miR-141-3p	hsa-miR-141	−17.64	1.08 × 10^−7^	2.18 × 10^−6^
hsa-miR-129-1-3p	hsa-miR-129-1	−17.21	7.85 × 10^−7^	7.91 × 10^−6^
hsa-miR-135a-5p	hsa-miR-135a-2	−16.31	4.17 × 10^−5^	1.77 × 10^−4^
hsa-miR-508-3p	hsa-miR-508	−16.13	3.20 × 10^−10^	4.68 × 10^−8^
hsa-miR-4461	hsa-miR-4461	−16.04	3.57 × 10^−10^	4.68 × 10^−8^
hsa-miR-885-5p	hsa-miR-885	15.65	6.08 × 10^−6^	3.46 × 10^−5^
hsa-miR-187-3p	hsa-miR-187	−14.12	4.83 × 10^−6^	2.88 × 10^−5^
hsa-miR-210-3p	hsa-miR-210	14.02	1.55 × 10^−13^	8.12 × 10^−11^
hsa-miR-210-5p	hsa-miR-210	13.47	1.38 × 10^−9^	1.26 × 10^−7^
hsa-miR-138-5p	hsa-miR-138-2	−12.09	2.01 × 10^−5^	9.40 × 10^−5^
hsa-miR-1251-5p	hsa-miR-1251	−10.98	3.74 × 10^−4^	1.08 × 10^−3^
hsa-miR-362-5p	hsa-miR-362	−9.99	7.65 × 10^−8^	2.04 × 10^−6^
hsa-miR-155-5p	hsa-miR-155	9.62	5.31 × 10^−8^	1.86 × 10^−6^

Principal component analysis (PCA) of differentially expressed miRNAs was used to segregate the samples according to their origin, i.e., tumour or normal tissue (Figure 1B). Tumour and normal samples were separated along the PC1 axis, accounting for 59.7% of the variation. The degree of variation appeared to be larger in the tumour group.

**Table 3:** Cox multivariate analyses for the GA (A) and HiSeq (B) datasets. miR-155 levels were used together with age and gender (Model 1) and age, gender, tumour stage and histological grade (Model 2).

**A**	**GA**			
	**Model 1**		**Model 2**	
	**HR (95% CI)**	***p***	**HR**	***p***
Per SD	1.18 (1.04, 1.35)	0.013	1.0 (0.82, 1.21)	0.97
Vs Q1				
Q2	1.88 (0.92, 3.83)	0.082	1.39 (0.7, 2.77)	0.342
Q3	2.19 (1.12, 4.28)	0.022	1.22 (0.62, 2.39)	0.567
Q4	3.03 (1.57, 5.85)	0.001	1.46 (0.76, 2.8)	0.261
Model 1	age, sex			
model 2	+stage, grade			
**B**	**HiSEQ**			
	**Model 1**		**Model 2**	
	**HR (95% CI)**	***p***	**HR**	***p***
Per SD	1.27 (1.00, 1.62)	0.046	1.1 (0.85–1.43)	0.449
Vs Q1				
Q2	0.98 (0.42, 2.27)	0.955	1.0 (0.42, 2.39)	0.996
Q3	1.84 (0.84, 4.01)	0.128	2.4 (1.07, 5.37)	0.033
Q4	1.67 (0.79, 3.53)	0.181	1.07 (0.5, 2.3)	0.862
Model 1	age, sex			
model 2	+stage, grade			

SD: standard deviation, Q1: quartile 1, HR: hazard ration, CI: confidence interval,

**Table 4:** Top affected pathways and upstream regulators. TGFβ1: Transforming growth factor beta 1, TNF: tumor necrosis factor, IFNG: Interferon gamma.

**Pathways**	***p*-Value**	**Overlap**
Th2 pathway	6.23 × 10^−11^	17.3% 26/150
Th1 and Th2 activation pathway	3.10 × 10^−10^	15.1% 28/185
Th1 pathway	3.12 × 10^−8^	15.6% 21/135
Antigen presentation pathway	9.02 × 10^−8^	28.9% 11/38
Hepatic fibrosis/hepatic stellate cell activation	3.88 × 10^−7^	12.6% 23/183
**Upstream Regulators**	***p*-Value**	
IFNG	3.41 × 10^−20^	
TNF	7.26 × 10^−16^	
Lipopolysaccharide TGG	2.01 × 10^−15^	
TGFβ1	1.03 × 10^−14^	
Beta-estradiol	1.12 × 10^−14^	

## Supplementary Materials

Supplementary materials can be found at http://www.mdpi.com/1422-0067/19/3/803/s1.

## Appendix A

**Table A1:** Top miRNAs from obesity-related inquiries.

	Top miRNAs	Abs. Fold Change	Adj. *p*-Value	
Tumour: BMI high vs. BMI low	miR-1251	10	0.440815	
	miR-483-3p	9.5	0.740509	
	mir-4792	8.7	0.151826	
	miR-146b	7.3	0.151826	
Normal: BMI high vs. low	mir-122	5.1	0.765518	
	miR-514a-5p	4.7	0.185964	
	miR-514a-3p	4.9	0.085238	
	34c-3p	3.9	0.765518	
BMI high: Tumour vs. Normal	miR-122-5p	280	6.78 × 10^−4^	
	miR-184	129	3.15 × 10^−5^	
	miR-122-3p	50	1.95 × 10^−4^	
	miR-891a-5p	42.9	3.87 × 10^−4^	
BMI low: Tumour vs. Normal	mir-184	310.1	1.56 × 10^−4^	
	mir-891a	137.5	2.19 × 10^−3^	
	mir-141	63.9	2.30 × 10^−4^	
	miR-122-5p	58.8	7.11 × 10^−4^	
Tumour (BMI high vs. low) vs. Normal (BMI high vs. low)	mir-483-3p	19.3	0.693293	
	mir-146b	10.6	3.20 × 10^−1^	
	mir-2277	9.9	3.20 × 10^−1^	
	miR-192-5p	6.1	4.39 × 10^−1^	

**Table A2:** Overview over which miRNAs were found to be most significant in which analysis.

miRNA	Amongst 20 Most Deregulated miRNAs in Our Dataset	Evaluated as Classifier	Amongst the 4 miRNA with the Strongest Correlation to Tumour Size	Amongst the top 4 miRNAs with the Strongest Correlation to Tumour Size	Found amongst the Top 17 miRNAs Found by Shu et al.	Found amongst the Significant Differentially Expressed miRNAs from the Work of Shu et al.	Found amongst the Top 20 miRNAs Found by Osanto et al.	Found amongst the Significant Differentially Expressed miRNAs Found by Osanto et al.	Survival Analysis Using TCGA Dataset	Survival Analysis Using Illumina GA Dataset
			(without Normal Samples)	(with Normal Samples)						
hsa-miR-122-5p	Yes	Yes	No	No	No	Yes	No	Yes	Yes	Yes
hsa-miR-885-5p	Yes	No	No	No	No	No	No	No	No	No
hsa-miR-210-3p	Yes	No	No	No	Yes	Yes	Yes	Yes	No	No
hsa-miR-210-5p	Yes	No	No	No	No	No	Yes	Yes	No	No
hsa-miR-138-5p	Yes	No	No	No	No	No	No	Yes	No	No
hsa-miR-187-3p	Yes	No	No	No	No	Yes	No	Yes	No	No
hsa-miR-4461	Yes	No	No	No	No	No	No	No	No	No
hsa-miR-508-3p	Yes	No	No	No	No	No	No	No	No	No
hsa-miR-135a-5p	Yes	No	No	No	No	Yes	No	Yes	No	No
hsa-miR-129-1-3p	Yes	No	No	No	No	Yes	No	Yes	No	No
hsa-miR-141-3p	Yes	No	No	No	Yes	Yes	No	Yes	No	No
hsa-miR-216b-5p	Yes	No	No	No	No	No	No	No	No	No
hsa-miR-514a-3p	Yes	No	No	No	No	Yes	No	No	Yes	Yes
hsa-miR-141-5p	Yes	No	No	No	No	Yes	No	No	No	No
hsa-miR-200c-3p	Yes	No	No	No	Yes	Yes	No	Yes	No	No
hsa-miR-891a-5p	Yes	No	No	No	No	No	Yes	yes	No	No
hsa-miR-184	Yes	Yes	No	No	No	No	No	No	Yes	Yes
hsa-miR-1304	No	No	Yes	No	No	No	No	No	No	No
hsa-miR-155	Yes	No	Yes	Yes	Yes	Yes	Yes	Yes	No	No
hsa-miR-142-3p	No	No	Yes	No	No	Yes	No	No	No	No
Hsa-miRNA-616-5p	No	No	Yes	No	No	No	No	No	No	No
Hsa-miRNA-361-3p	No	No	No	Yes	No	No	No	No	No	No
Hsa-miRNA-10b-3p	No	No	No	Yes	No	No	No	No	No	No
Hsa-miRNA-10b-5p	No	No	No	Yes	Yes	Yes	No	Yes	No	No
Hsa-miRNA-146	No	No	No	No	No	Yes	No	Yes	Yes	Yes
Hsa-miRNA-362	Yes	No	No	No	No	Yes	No	Yes	No	No
Hsa-miRNA-1251	Yes	No	No	No	No	No	No	No	No	No
